# Is it time to re-evaluate the ban on body checking in women's ice hockey as a key parameter for reducing the incidence of concussions?

**DOI:** 10.3389/fspor.2026.1804886

**Published:** 2026-06-11

**Authors:** Mikael Swarén

**Affiliations:** Swedish Unit for Metrology in Sports, Department of Sports and Health Sciences, School of Health and Welfare, Dalarna University, Falun, Sweden

**Keywords:** contact sports, elite sports, female, head impact, regulations

## Abstract

This perspective report examines temporal trends in concussion rates in relation to changes in permitted physical contact in the Swedish Women's Hockey League (SDHL). Over three seasons, the league gradually allowed more physical play, culminating in the current permission of body checking from the 2022/2023 season. Contrary to traditional assumptions, reported concussion rates declined during a period of progressively increased permitted physical contact. However, these observational findings should be interpreted with caution, as multiple contextual factors may have influenced the results. Also, the results do not establish a causal relationship between body checking policy and concussion incidence and rather than supporting immediate policy change, the findings highlight the need for continued monitoring and further research into how rules, education, and player behavior interact to influence concussion risk.

## Introduction

For several years, the high incidence of concussions in ice hockey has been a subject of international attention and numerous studies have been conducted to mitigate the occurrence of concussions in ice hockey, as well as exposure differences between male and female players (1–5). Even though women's ice hockey does not permit deliberate contacts (checking) between players. Earlier studies highlight that female players may experience higher concussion rates and symptom burden compared with male players, although the underlying reasons remain unclear and contemporary comparisons are limited (6, 7).

Mihalik et al. (2) investigated head impact exposure among youth ice hockey players (girls and boys, age 13–16 years where boys are allowed to bodycheck but girls are not) and showed that girls sustained fewer impacts compared to boys (13 impacts per player per season vs. 264 impacts per player per season), but with slightly higher median linear accelerations (18.1 g vs. 17.1 g). No significant difference between boys and girls regarding rotational accelerations was observed. These findings are in line with results by Eckner et al. (8) who compared head impact exposure between male and female high school ice hockey players and found that male players, who are allowed to bodycheck, experience more head impacts compared to females (7.7 impacts per player per game vs. 5.3 impacts per player per game), whereas the mean impact magnitudes were greater for the female players (18.8 g vs. 17.1 g). Furthermore, female players experienced more impacts to the side and top of the head compared with male players who had more impacts to the front and back (8).

In 2018, the Swedish ice hockey federation launched the “No concussion project”, to investigate the prevalence of concussions in the Swedish Women's Hockey League (SDHL). The analyses presented in this perspective paper are based on prospectively collected concussion registry data from the SDHL. Since the 2018/2019 season, all teams in the SDHL have been required to report all diagnosed concussions. The diagnosis of concussions and the reporting to the registry are handled by the medical officer in each team. Furthermore, all SDHL teams are mandated to administer ImPACT baseline testing as part of the league's compulsory medical protocol (9). The resulting baseline data are subsequently utilised to inform post-concussion assessments and to guide return-to-play decision-making.

## Bodychecking and concussions in the Swedish women's hockey league

During the 2018/2019 regular season, the SDHL had 35 reported concussions (194 concussions per 1000 game exposures), which is a concussion incidence rate equivalent to that of the men's highest league, the Swedish Hockey League (SHL), with 1.6 concussions per matchday. This was despite rule restrictions limiting body checking in women's hockey. Previous studies have not been able to identify why female players have such high concussion and head impact rates. Swarén and Fahlstedt (3) hypothesise that some head impacts among professional female ice hockey players may result from their ability to focus primarily on the puck, as they are not required to actively scan the rink to anticipate body checks, given that checking is prohibited in women's ice hockey. In an earlier study (10), the authors speculated that a potential risk factor for female hockey players might be their lack of experience and training in receiving and delivering body checks, due to the prohibition of checking in the women's game, which could leave them less prepared for incidental contact.

**Figure 1:**
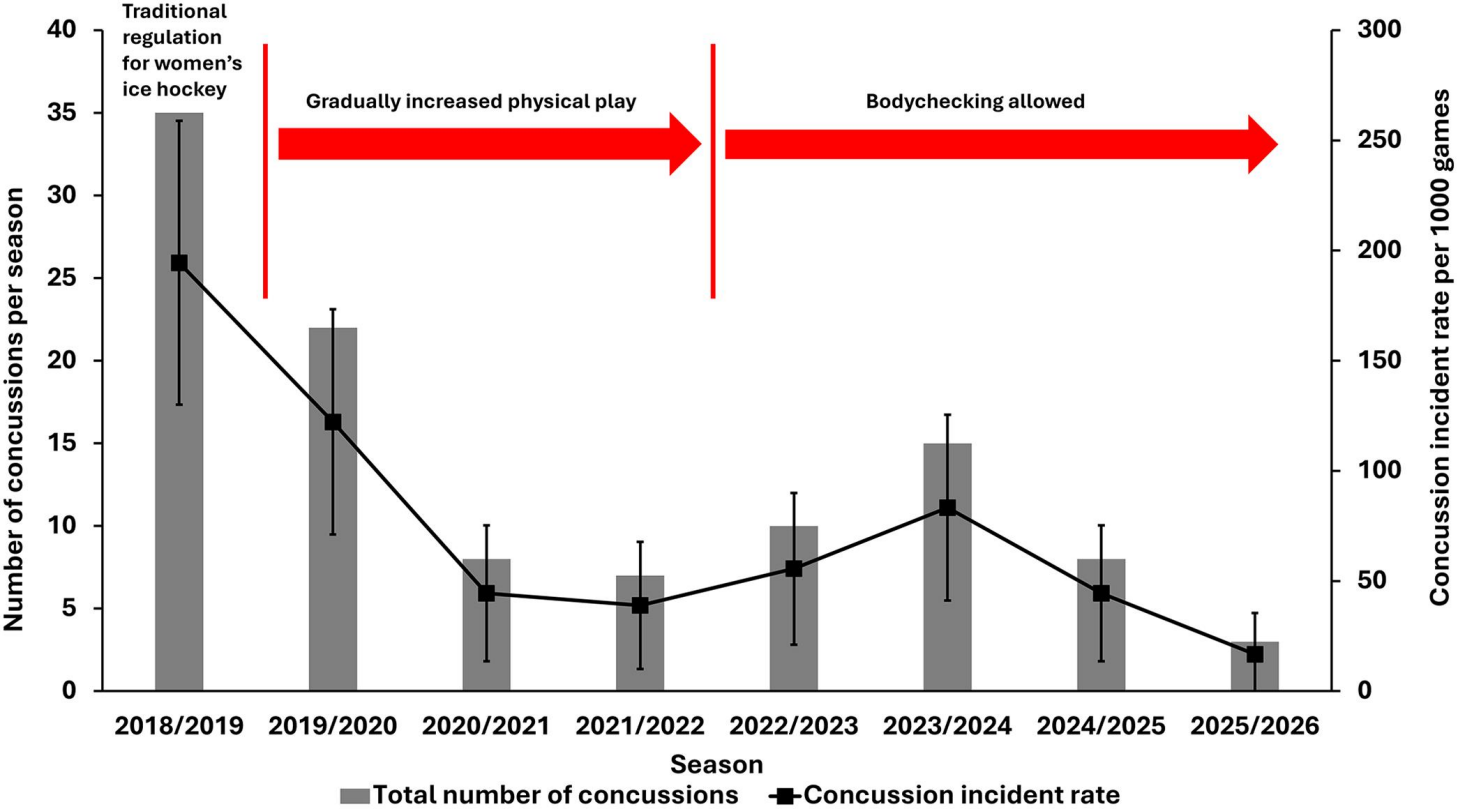
Number of reported and confirmed concussions in the SDHL, during the regular seasons (left axis) and concussion incidence per 1000 games per season with 95% confidence intervals (right axis). The 2018/2019 season employed the regular rule book for women's ice hockey which does not allow body checks. From the 2019/2020 season, increased body contact was allowed, including using the boards in battles for puck possession during the 2020/2021 season and allowing stationary players to engage in close combat, and enabling players to actively seek body contact during the 2021/2022 season. For the 2022/2023 season, body checks were allowed in the SDHL.

In response to this, the SDHL, in collaboration with the Swedish ice hockey federation, SDHL referees, the players’ association and team coaches, decided to permit increased physical contact between players starting in the 2019/2020 season. Body checking was prohibited as a baseline during the 2018/2019 season, with stepwise increases in permitted physical contact introduced prior to each season through rule modifications, and no within-season changes applied. The first policy change for the 2019/2020 season allowed players to anticipate physical contact while competing for the puck, even along the boards. That season, the number of concussions dropped from 35 in the 2018/2019 season to 22 in 2019/2020. Hence, the SDHL further expanded the physical contact regulations for the 2020/2021 season, permitting players to use the boards in battles for puck possession. Before the 2021/2022 season, additional physical contact was authorised, allowing stationary players to engage in close combat, and enabling players to actively seek body contact. This increase in permitted body contact did not lead to a rise in concussions, and given the marked decrease in concussions in the SDHL despite increased physical contact, SDHL and the Swedish Ice Hockey Association decided, as the first nation worldwide, to officially allow body checking in women's ice hockey for the 2022/2023 season. The only exception to the men's ice hockey rule book was the prohibition of “north-south hits”, where two players skating directly toward each other deliver open-ice body checks, as these carry a high risk of injury, even in men's hockey. All confirmed concussions and concussion incidence rates with 95% confidence intervals in the SDHL for the last eight regular seasons are presented in Figure 1. All regular seasons consisted of 10 teams, and a total of 180 games, resulting in a reduction from 194 concussions per 1000 game exposures during the 2018/2019 season, to 17 concussions per 1000 game exposures, during the 2025/2026 season.

## Complexities of concussion risk

Importantly, the prohibition of body checking does not necessarily imply the absence of physical contact during gameplay. Body contact still occurs frequently despite rule restrictions, influenced by factors such as refereeing standards, player behavior, and the operational definitions of illegal contact. Therefore, the observed reduction in concussion rates in the SDHL cannot be interpreted as a direct effect of allowing body checking *per se*. It is plausible that increased awareness, improved education, and enhanced preparedness for physical contact contributed to safer player behavior and reduced injury risk. Previous research within boys youth ice hockey (11–13) has shown a reduction in concussions when disallowing body checking in youth and adolescent ice hockey. At the youth level, where players develop fundamental hockey and contact-related skills, body checking is typically restricted or introduced progressively, and the present findings suggest that emphasis on education, anticipation, and controlled exposure to contact may be as important as rule restrictions in mitigating concussion risk. However, it remains unclear whether the observed trends are attributable to the introduction of body checking itself or to the increased emphasis on contact education and preparedness, suggesting that these factors likely interact and should be considered jointly in injury prevention strategies.

The current results highlight the complex relationship between body checking and concussion incidence in women's ice hockey. Contrary to previous assumptions, the introduction of body checking did not lead to an increase in concussions; instead, the number of reported concussions decreased. The observed trends may reflect a complex interplay of factors, including changes in player awareness, education, and adaptation to evolving game conditions. While it is possible that increased exposure to structured physical contact influences player behavior, the present data do not allow for conclusions regarding underlying mechanisms. While illegal hits certainly can result in concussions, head injuries can also occur in isolated incidents where a player falls independently, far from any opponent. Ice hockey inherently carries an elevated risk of concussions (and other injuries) due to the high speeds, hard boards surrounding the rink, and the potential impact of a puck traveling up to 160 km/h. However, maintaining high situational awareness, proper checking technique, and respect for opponents is crucial in minimising unnecessary injury risks.

## Concussion reporting considerations

Caution is warranted when interpreting reported data, as variations in reporting bias and willingness to report may occur between teams and across seasons. It can be hypothesised that the number of identified and documented concussions would typically increase following the initiation of a project in which teams are mandated to detect and report such injuries, contrary to what has been observed in the present SDHL data. Nevertheless, given that concussion constitutes a serious medical condition, team medical officers are required to record and report these cases in both medical journals and official reports, which supports a high degree of reliability in the available data. Also, the study period overlaps with the COVID-19 pandemic, which may have influenced player exposure, match intensity, medical reporting practices, and overall participation. These factors should be considered when interpreting temporal trends in concussion incidence. However, the possibility of increased risk awareness or evolving attitudes toward injury reporting among players cannot be excluded as contributing factors to the observed reduction in reported concussions. Still, the results challenge the traditional view that banning body checking is necessary for reducing concussion incidence in women's ice hockey. Lahti et al. (14) further support this perspective, reporting that 88% of SDHL players are in favor of allowing checking, with 64% believing it would not increase injury risks overall. Importantly, body checking may still occur despite being prohibited, and differences in refereeing awareness or interpretation could influence both enforcement and reporting, limiting the ability to draw conclusions without detailed gameplay analysis.

## Discussion

Although the present findings should be interpreted within the constraints of an observational, descriptive analysis of temporal trends, they challenge the prevailing assumption that restricting body checking is necessary to protect female ice hockey players from concussions. The observed reduction in reported concussions following the gradual introduction of body checking in the SDHL suggests that exposure to controlled physical contact may influence player awareness and preparedness in ways that are not fully captured by current norms. However, even though a reduction in reported concussions was observed during a period of regulatory change, no causal inferences can be drawn regarding the relationship between body checking policies and concussion incidence. Future research using controlled or comparative designs is needed to better understand how rule changes, player behavior, and the risk of concussion are associated. Until then, conclusions should remain descriptive, and policy implications should be considered with caution. While this report focuses on concussions, body checking is also a well-established risk factor for overall injury burden in ice hockey. Future studies should therefore adopt a broader injury perspective to better capture the overall health impact of rule changes. Rather than drawing definitive conclusions, these results highlight the need to continue to critically monitor and evaluate regulatory approaches in women's ice hockey and to encourage further research into how rules, training, and playing experience interact to influence concussion incidence and overall injury risk, across professional, amateur, and youth ice hockey.

## Data Availability

Publicly available datasets were analyzed in this study. These data can be found here: https://www.sdhl.se/kontakt.
